# The Associations between Asymmetric Handgrip Strength and Chronic Disease Status in American Adults: Results from the National Health and Nutrition Examination Survey

**DOI:** 10.3390/jfmk6040079

**Published:** 2021-09-23

**Authors:** Lukus Klawitter, Adam Bradley, Kyle J. Hackney, Grant R. Tomkinson, Bryan K. Christensen, William J. Kraemer, Ryan McGrath

**Affiliations:** 1 Department of Health, Nutrition, and Exercise Sciences, North Dakota State University, Fargo, ND 58108, USA; lukus.klawitter@ndsu.edu (L.K.); adam.bradley@ndsu.edu (A.B.); kyle.hackney@ndsu.edu (K.J.H.); bryan.christensen.1@ndsu.edu (B.K.C.); 2 Department of Education, Health and Behavior Studies, University of North Dakota, Grand Forks, ND 58202, USA; grant.tomkinson@und.edu; 3 Alliance for Research in Exercise, Nutrition and Activity, School of Health Sciences, University of South Australia, Adelaide, SA 5501, Australia; 4 Department of Human Sciences, The Ohio State University, Columbus, OH 43210, USA; Kraemer.44@osu.edu; 5 Fargo VA Healthcare System, Fargo, ND 58102, USA

**Keywords:** comorbidity, chronic disease, epidemiologic research design, muscle strength, screening

## Abstract

This study examined the associations between asymmetric handgrip strength (HGS) and multimorbidity in American adults. Secondary analyses of data from persons aged at least 40 years from the 2011–2012 and 2013–2014 waves of the National Health and Nutrition Examination Survey were conducted. A handheld dynamometer collected HGS on each hand and persons with a strength imbalance >10% between hands were classified as having asymmetric HGS. Adults with the presence of ≥2 of the following conditions had multimorbidity: cardiovascular disease, chronic obstructive pulmonary disease, chronic kidney disease, asthma, arthritis, cancer, obesity, stroke, hypertension, high cholesterol, and diabetes. Of the *n* = 3483 participants included, *n* = 2700 (77.5%) had multimorbidity. A greater proportion of adults with multimorbidity had HGS asymmetry (*n* = 1234 (45.7%)), compared to persons living without multimorbidity (*n* = 314 (40.1%); *p* < 0.05). Relative to individuals without asymmetry, adults with asymmetric HGS had 1.31 (95% confidence interval (CI): 1.03–1.67) greater odds for multimorbidity. Moreover, persons with HGS asymmetry had 1.22 (CI: 1.04–1.44) greater odds for accumulating morbidities. Asymmetric strength, as another indicator of diminished muscle function, is linked to chronic morbidity status. Healthcare providers should recommend healthy behaviors for reducing asymmetries to improve muscle function and mitigate morbidity risk after completing asymmetry screenings.

## 1. Introduction

Handgrip dynamometers are frequently utilized to collect handgrip strength (HGS) in clinical settings, translational research, and population-based studies [1]. Measures of HGS simply and reliably assess overall muscle strength [2]. Declines in HGS are associated with several chronic cardiometabolic conditions and early all-cause mortality [3,4]. Although handgrip dynamometers are a feasible and viable screening tool for morbidities linked to low strength capacity, HGS has limited utility because strength capacity alone does not coalesce the array of physical characteristics that underpin muscle function [5]. Therefore, evaluating methods for improving muscle function screening while maintaining feasibility may elevate the prognostic value of muscle function assessments.

Current HGS protocol guidelines suggest that multiple trials of HGS be collected on each hand, and the greatest HGS value, regardless of hand dominance, be included as maximal HGS [6]. However, bilateral strength asymmetry has been identified as a factor that may influence health [7], and HGS protocol guidelines already allow for strength asymmetries to be conveniently evaluated because maximal isometric grip force is collected on each hand in multiple trials [6].

Asymmetric strength may represent another characteristic of muscle dysfunction before deficiencies in overall strength capacity are observed [8]. Previous investigations have revealed that asymmetric HGS is associated with diminished health and shortened longevity [9,10]. Yet, it is largely unknown how asymmetric strength may factor into populations that are likely to have poor muscle function such as those living with multiple chronic cardiometabolic morbidities. Examining strength asymmetries in such populations may help improve muscle function screenings for individuals at risk for morbid conditions and allow for relevant muscle strengthening interventions to restore strength balance between limbs. The purpose of this study was to determine the associations between HGS asymmetry and multimorbidity in a national sample of American adults.

## 2. Materials and Methods

### 2.1. Participants

We conducted a secondary analysis of cross-sectional data from the 2011–2012 and 2013–2014 waves of the National Health and Nutrition Examination Survey (NHANES). These NHANES waves were selected because they included HGS measurements. The NHANES is designed to observe health factors in Americans [11]. Interviewers collected health-related information in respondents’ homes and study participants also visited mobile examination centers for additional assessments. The NHANES diversified sampling by traveling to multiple locations throughout the United States [12]. Oversampling occurred in older adults and persons identifying as Hispanic, non-Hispanic Asian, and non-Hispanic Black to generate representative data [11]. Overall response rates for the NHANES were generally high for each of the waves we analyzed [13].

The analytic sample included Americans aged ≥40 years with information for HGS and relevant self-report measures. Our age criterion for inclusion was informed by other NHANES studies that have utilized this age threshold for categorizing adults as at least middle-aged [14,15]. Participants gave written informed consent before engaging in the NHANES, and protocols were approved by the National Center for Health Statistics Research Ethics Review Board (Protocol #2011-17).

### 2.2. Measures

#### 2.2.1. Multimorbidity

Height was collected with a stadiometer and body mass was measured with an electronic scale (Mettler-Toledo International, Inc.; Columbus, OH, USA) using routine protocols. Body mass index (BMI) was calculated as kilograms per meters-squared (kg/m^2^) and persons considered obese had a BMI of ≥30 kg/m^2^ [14]. Respondents told interviewers if a doctor had diagnosed them with cardiovascular disease, chronic lung disease, chronic kidney disease, asthma, arthritis, cancer, stroke, high blood pressure, high cholesterol, and diabetes. The presence of obesity and the reported health conditions for determining multimorbidity were chosen because of their clinical relevance and availability in the NHANES [16]. Persons with multimorbidity had ≥2 chronic conditions.

#### 2.2.2. Handgrip Strength Asymmetry

HGS was collected with a handgrip dynamometer (Takei Dynamometer Model T.K.K.5401; Akiha-Ku, Japan). All interviewers were provided training for the HGS procedures and a calibration manual. Individuals unable to grasp the dynamometer on an individual hand did not complete HGS testing on that hand. Before HGS testing, participants were asked about their ability to complete the HGS protocols, including if they had a procedure within the past three months that would limit their testing abilities. All HGS procedures were explained and demonstrated by the trained interviewers.

Participants indicated their dominant hand (right, left), and the decision to start HGS testing on the right or left hand was randomized. The dynamometer was fitted to the hand size of participants and a sub-maximal practice trial was completed to verify the interpretation of the HGS procedures. Participants squeezed the dynamometer at maximal effort for each trial. The right and left hands were tested three times, alternating hands between trials, with 1 min of rest between measurements on the same hand. More details about the HGS procedures used in the NHANES are available elsewhere [17,18]. Participants with HGS on each hand were included in our analyses, and the greatest HGS value on each hand was used in the calculation for asymmetry.

While HGS differs and is linked to hand dominance, the “10% rule” [19], which posits the strength of the dominant hand is about 10% greater than the non-dominant hand, was utilized to guide how we defined HGS asymmetry. Studies examining asymmetric HGS have also utilized a 10% HGS asymmetry cut point [9,10]. HGS asymmetry ratio was calculated from the highest recorded HGS values from each hand: (non-dominant HGS (kilograms) / dominant HGS (kilograms)). Persons with asymmetric HGS greater than 10% in either direction (i.e., ratio <0.90 or >1.10) had HGS asymmetry.

#### 2.2.3. Covariates

Age, marital status, educational achievement, and cigarette smoking status were self-reported. Participants also identified their sex and ethnicity. A five-item self-rated health indicator was used to characterize perceived health. The single highest recorded HGS value was utilized for defining weakness. Males and females with maximal HGS <26-kilograms and <16-kilograms were considered weak, respectively [20]. Depression status was examined with the nine-item patient health questionnaire [21]. Individuals with scores of ≥10 were classified as having depression [21].

Participants reported their abilities to perform 19 tasks from 5 different functional categories. The functional categories included: (1) basic activities of daily living (“getting in and out of bed”, “using a fork, knife, and cup”, “walking between rooms on the same floor”, “dressing yourself”); (2) instrumental activities of daily living (“house chores”, “managing money”, “preparing meals”); (3) leisure and social activities (“going out to movies and events”, “leisure activities at home”, “attending social events”); (4) lower extremity mobility (“walking up 10 steps”, “walking for a quarter mile”); (5) general physical tasks (“grasping or holding small objects”, “lifting or carrying”, “reaching up and overhead”, “sitting for long periods of time”, “standing for long periods of time”, “standing up from an armless chair”, “stooping, crouching, kneeling”). Persons were classified as having a functional disability if they indicated having “some difficulty”, “much difficulty”, or an “inability” to complete any of the functional tasks. Previous investigations have utilized these criteria for operationalizing functional abilities with NHANES data [22,23].

### 2.3. Statistical Analysis

All analyses were performed with SAS 9.4 software (SAS Institute; Cary, NC, USA). Independent *t*-tests (continuous variables) and chi-squared tests (categorical variables) examined differences in the participant characteristics for persons living with and without multimorbidity. Crude and fully adjusted logit regression models analyzed the associations between HGS asymmetry (reference: HGS asymmetry ≤10%) and multimorbidity. Moreover, crude and fully adjusted ordinal logistic regression models were utilized to quantify the associations between HGS asymmetry (reference: HGS asymmetry ≤10%) and each accumulating morbidity. The fully adjusted models included age, sex, ethnicity, weakness, hand dominance, educational achievement, self-rated health, marital status, cigarette smoking status, depression status, and functional disability as covariates. The findings from our fully adjusted models were considered our primary results.

To examine the role of sex as a biological variable, the fully adjusted logistic, and ordinal logit regression models were stratified by sex. The sex-stratified analyses were presented as supplementary. Sample weights provided by the NHANES were utilized in our models to acknowledge the complex sampling methods and generate an unbiased national-representative estimate [24]. An alpha level of 0.05 was used for the analyses.

## 3. Results

The demographic characteristics of the participants are presented in Table 1. Overall, the *n* = 3483 participants were aged 65.6 ± 10.3 years and *n* = 2700 had multimorbidity (77.5%). A higher proportion of HGS asymmetry (*n* = 1234 (45.7%)) was observed in persons with multimorbidity compared to adults without multimorbidity (*n* = 314 (40.1%); *p* < 0.05).

**Figure 1:**
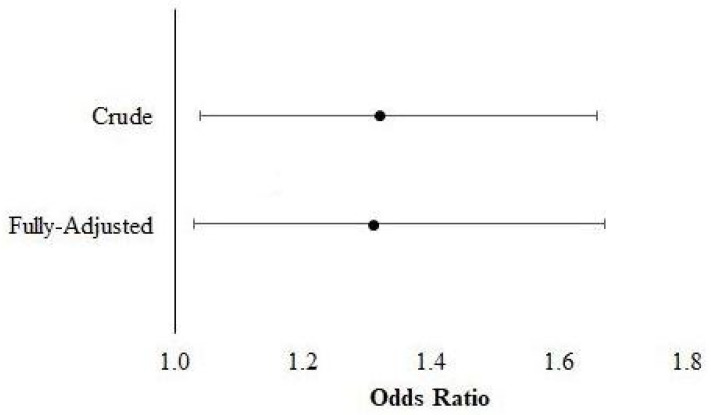
Results for the association between handgrip strength asymmetry and multimorbidity.

**Figure 2:**
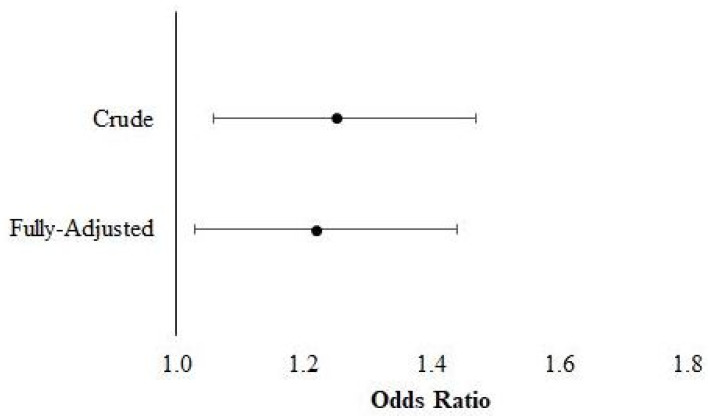
Results for the association between handgrip strength asymmetry and accumulating morbidities.

Figure 1 shows the findings for the associations between asymmetric HGS and multimorbidity. The crude analyses suggest that persons with asymmetric HGS had 1.32 (95% confidence interval (CI: 1.05, 1.66) greater odds for multimorbidity, while the fully adjusted estimates indicate that adults with HGS asymmetry had 1.31 (CI: 1.03, 1.67) greater odds for multimorbidity. The results for the associations between asymmetric HGS and accumulating morbidities are depicted in Figure 2. The crude and fully adjusted analyses likewise suggest that Americans with HGS asymmetry had 1.25 (CI: 1.06, 1.47) and 1.22 (CI: 1.04, 1.44) greater odds for each additional morbidity, respectively.

Table A1 shows the results for the associations between asymmetric HGS and multimorbidity by sex, while Table A2 presents the results for the associations between asymmetric HGS and accumulating morbidities by sex. Women with HGS asymmetry had 1.42 (CI: 1.01, 1.99) greater odds for multimorbidity and 1.26 (CI: 1.01, 1.57) greater odds for accumulating morbidities. No other statistically significant associations were observed for these sex-stratified analyses.

## 4. Discussion

The overarching findings of our study revealed that asymmetric HGS was associated with multimorbidity status in American adults. Specifically, persons with asymmetric HGS had 31% greater odds for multimorbidity and 22% greater odds for each accumulating morbidity. These findings indicate that strength asymmetries are associated with multiple chronic cardiometabolic conditions. Healthcare providers should consider utilizing handgrip dynamometers as a simple, non-invasive method for examining strength asymmetries. Referrals to appropriate muscle-strengthening activities are advised for persons identified as having asymmetric strength in an effort to regain functional symmetry between limbs and mitigate morbidity risk.

Our findings align with the results from previous studies that suggest HGS asymmetry is associated with poor health and shortened longevity [9,10]. Engaging in unhealthy lifestyle behaviors such as physical inactivity may contribute to poor muscle quality and function that leads to elevated morbidity risk [25,26]. For example, the association between HGS asymmetry and multimorbidity could be explained by skeletal muscle impairments observed in persons with diabetes neuropathy [27]. Interventions that include muscle-strengthening activities may help to improve functional asymmetries in persons identified as having asymmetric strength. Although it is important to consider each person’s functional abilities, utilizing the physical activity guidelines for Americans may serve as a reference point for healthcare providers and their applicable patients [28]. Moreover, relevant interventions aiming to improve muscle function, including strength asymmetries, may consider evaluating HGS asymmetry for determining intervention effectiveness given that maximal HGS alone has demonstrated marginal efficacy [29].

Diversifying our assessments of muscle function may help to uncover risk factors for poor health that may not be otherwise identified by strength capacity alone. For example, a similar investigation that utilized NHANES data found that mean combined relative HGS was associated with metabolic syndrome in American adults [30]. Further, our findings parallel those from Liu et al. [31], wherein was found that HGS asymmetry was linked to multimorbidity, especially in women, with data from the English Longitudinal Study of Aging. Therefore, we suggest that asymmetry be measured alongside maximal strength in applicable HGS protocols and considered in secondary analyses with data such as NHANES because maximal HGS is already collected on both hands. Further advancing how muscle function is assessed may improve screening for muscle dysfunction and subsequent disease risk. Advancements may include reevaluating HGS protocols, utilizing modern HGS technologies, and examining different muscle function aspects aside from maximal strength alone for health and human performance in different populations.

Our study was not without limitations. A cross-sectional design was employed by the NHANES, which creates problems related to the direction of causation for our findings. While self-report is common in epidemiological survey studies such as NHANES, self-report biases may have impacted how participants identified their morbidity status. Morbidities that were not available in the NHANES could not be examined in our analyses. Some of the morbidities included in our analyses may have a higher prevalence and be influenced by strength asymmetry more than others. Although we conducted ordinal logistic regression models, certain morbidities included in our analyses are strongly related to other morbidities we examined. The “10% rule” guided how we determined asymmetry, but other thresholds for determining asymmetry may exist. The development of more standardized cut points for defining HGS asymmetry is warranted. While the number of ambidextrous persons might be smaller, examining asymmetry in ambidextrous individuals may reveal unique insights into asymmetry and health. We conducted secondary analyses of the 2011–2012 and 2013–2014 waves of the NHANES. Indeed, the NHANES is a rich data source, but the waves we evaluated could be considered dated by some. Nonetheless, the NHANES provides powerful, nationally representative data with procedures and measures that have modern-day applicability.

## 5. Conclusions

Our findings revealed that American adults with HGS asymmetry had greater odds for multimorbidity and accumulating morbidities. We recommend that healthcare providers utilize handgrip dynamometers as a feasible method for assessing strength asymmetries between limbs. Persons identified as having asymmetric strength should be referred to relevant programs that foster muscle-strengthening activities that help to correct functional asymmetries. Participation in such programs may help lower multimorbidity risk in American adults.

## Figures and Tables

**Table 1:** Descriptive characteristics of the participants.

Variable	Overall (*n* = 3483)	No Multimorbidity (*n* = 783)	Multimorbidity (*n* = 2700)
Age (years)	65.6 ± 10.3	63.3 ± 10.2	66.3 ± 10.2 *
Maximal handgrip strength (kilograms)	32.7 ± 10.5	34.6 ± 10.6	32.1 ± 10.4 *
Handgrip strength asymmetry (n (%))	1548 (44.4)	314 (40.1)	1234 (45.7) *
Right hand dominant (n (%))	3202 (91.9)	726 (92.7)	2476 (91.7)
Reported morbidities	3.0 ± 1.8	0.7 ± 0.5	3.6 ± 1.4 *
Weak (n (%))	178 (5.1)	33 (4.2)	145 (5.4)
Female (n (%))	1825 (52.4)	342 (43.7)	1483 (54.9) *
Non-Hispanic white (n (%))	1683 (48.3)	450 (57.5)	1350 (50.0) *
Excellent, very good, or good self-rated health (n (%))	2356 (67.6)	613 (78.3)	1743 (64.6) *
Current smoker (n (%))	618 (17.7)	165 (21.1)	453 (16.8) *
Married (n (%))	1806 (51.9)	414 (52.9)	1392 (51.6)
Depressed (n (%))	441 (12.7)	64 (8.2)	377 (14.0) *
Not a high school graduate (n (%))	920 (26.4)	208 (26.6)	712 (26.4)
Functional disability (n (%))	2390 (68.6)	407 (52.0)	1983 (73.4) *

Results are reported as mean ± standard deviation or frequency (percentage) where indicated. * *p* < 0.05.

## Data Availability

National Health and Nutrition Examination Survey data are publicly available online at: https://www.cdc.gov/nchs/nhanes/index.htm (accessed on 27 July 2021).

## Appendix A

**Table A1:** Associations between handgrip strength asymmetry and multimorbidity by sex.

Variable	Fully Adjusted Odds Ratio (95% Confidence Interval)
Handgrip Strength Asymmetry	
Men	1.23 (0.87, 1.73)
Women	1.42 (1.01, 1.99)

**Table A2:** Associations between handgrip strength asymmetry and accumulating morbidities by sex.

Variable	Fully Adjusted Odds Ratio (95% Confidence Interval)
Handgrip Strength Asymmetry	
Men	1.23 (0.96, 1.58)
Women	1.26 (1.01, 1.57)
